# CONSORT 2025 statement: updated guideline for reporting randomised trials

**DOI:** 10.1136/bmj-2024-081123

**Published:** 2025-04-14

**Authors:** Sally Hopewell, An-Wen Chan, Gary S Collins, Asbjørn Hróbjartsson, David Moher, Kenneth F Schulz, Ruth Tunn, Rakesh Aggarwal, Michael Berkwits, Jesse A Berlin, Nita Bhandari, Nancy J Butcher, Marion K Campbell, Runcie C W Chidebe, Diana Elbourne, Andrew Farmer, Dean A Fergusson, Robert M Golub, Steven N Goodman, Tammy C Hoffmann, John P A Ioannidis, Brennan C Kahan, Rachel L Knowles, Sarah E Lamb, Steff Lewis, Elizabeth Loder, Martin Offringa, Philippe Ravaud, Dawn P Richards, Frank W Rockhold, David L Schriger, Nandi L Siegfried, Sophie Staniszewska, Rod S Taylor, Lehana Thabane, David Torgerson, Sunita Vohra, Ian R White, Isabelle Boutron

**Affiliations:** 1 Oxford Clinical Trials Research Unit, Centre for Statistics in Medicine, University of Oxford, Oxford OX3 7LD, UK; 2 Department of Medicine, Women’s College Research Institute, University of Toronto, Toronto, ON, Canada; 3 UK EQUATOR Centre, Centre for Statistics in Medicine, University of Oxford, Oxford, UK; 4 Centre for Evidence-Based Medicine Odense and Cochrane Denmark, Department of Clinical Research, University of Southern Denmark, Odense, Denmark; 5 Open Patient data Explorative Network, Odense University Hospital, Odense, Denmark; 6 Centre for Journalology, Clinical Epidemiology Programme, Ottawa Hospital Research Institute, Ottawa, ON, Canada; 7 Department of Obstetrics and Gynecology, School of Medicine, University of North Carolina at Chapel Hill, Chapel Hill, NC, USA; 8 Jawaharlal Institute of Postgraduate Medical Education and Research, Puducherry, India; 9 Office of Science Dissemination, Centers for Disease Control and Prevention, Atlanta, GA, USA; 10 Department of Biostatistics and Epidemiology, School of Public Health, Center for Pharmacoepidemiology and Treatment Science, Rutgers University, New Brunswick, NJ, USA; 11 *JAMA Network Open*, Chicago, IL, USA; 12 Centre for Health Research and Development, Society for Applied Studies, New Delhi, India; 13 Child Health Evaluation Services, The Hospital for Sick Children Research Institute, Toronto, ON, Canada; 14 Department of Psychiatry, University of Toronto, Toronto, ON, Canada; 15 Aberdeen Centre for Evaluation, University of Aberdeen, Aberdeen, UK; 16 Project PINK BLUE - Health & Psychological Trust Centre, Utako, Abuja, Nigeria; 17 Department of Sociology and Gerontology, Miami University, OH, USA; 18 Department of Medical Statistics, London School of Hygiene and Tropical Medicine, London, UK; 19 Nuffield Department of Primary Care Health Sciences, University of Oxford, Oxford, UK; 20 Ottawa Hospital Research Institute, Ottawa, ON, Canada; 21 Department of Medicine, Northwestern University Feinberg School of Medicine, Chicago, IL, USA; 22 Department of Epidemiology and Population Health, Stanford University, Palo Alto, CA, USA; 23 Institute for Evidence-Based Healthcare, Faculty of Health Sciences and Medicine, Bond University, University Drive, Robina, QLD, Australia; 24 Departments of Medicine, of Epidemiology and Population Health, of Biomedical Data Science, and of Statistics, and Meta-Research Innovation Center at Stanford (METRICS), Stanford University, Stanford, CA, USA; 25 MRC Clinical Trials Unit at University College London, London, UK; 26 University College London, UCL Great Ormond Street Institute of Child Health, London, UK; 27 NIHR Exeter Biomedical Research Centre, Faculty of Health and Life Sciences, University of Exeter, Exeter, UK; 28 Edinburgh Clinical Trials Unit, Usher Institute-University of Edinburgh, Edinburgh BioQuarter, Edinburgh, UK; 29 *The BMJ*, BMA House, London, UK; 30 Harvard Medical School, Boston, MA, USA; 31 Université Paris Cité, Inserm, INRAE, Centre de Recherche Epidémiologie et Statistiques, Université Paris Cité, Paris, France; 32 Clinical Trials Ontario, MaRS Centre, Toronto, ON, Canada; 33 Duke Clinical Research Institute, Duke University Medical Center, Durham, NC, USA; 34 Department of Emergency Medicine, University of California, Los Angeles, CA, USA; 35 South African Medical Research Council, Cape Town, South Africa; 36 Warwick Applied Health, Warwick Medical School, University of Warwick, Coventry, UK; 37 MRC/CSO Social and Public Health Sciences Unit & Robertson Centre for Biostatistics, Institute of Health and Wellbeing, University of Glasgow, Glasgow, UK; 38 Department of Health Research Methods Evidence and Impact, McMaster University, Hamilton, ON, Canada; 39 St Joseph’s Healthcare Hamilton, Hamilton, ON, Canada; 40 York Trials Unit, Department of Health Sciences, University of York, York, UK; 41 Faculty of Medicine and Dentistry, University of Alberta, Edmonton, AB, Canada; 42 Université Paris Cité and Université Sorbonne Paris Nord, Inserm, INRAE, Centre for Research in Epidemiology and Statistics (CRESS), Paris, France; 43 Centre d’Epidémiologie Clinique, Hôpital Hôtel Dieu, AP-HP, Paris, France

## Abstract

**Background:**

Well designed and properly executed randomised trials are considered the most reliable evidence on the benefits of healthcare interventions. However, there is overwhelming evidence that the quality of reporting is not optimal. The CONSORT (Consolidated Standards of Reporting Trials) statement was designed to improve the quality of reporting and provides a minimum set of items to be included in a report of a randomised trial. CONSORT was first published in 1996, then updated in 2001 and 2010. Here, we present the updated CONSORT 2025 statement, which aims to account for recent methodological advancements and feedback from end users.

**Methods:**

We conducted a scoping review of the literature and developed a project-specific database of empirical and theoretical evidence related to CONSORT, to generate a list of potential changes to the checklist. The list was enriched with recommendations provided by the lead authors of existing CONSORT extensions (Harms, Outcomes, Non-pharmacological Treatment), other related reporting guidelines (TIDieR) and recommendations from other sources (eg, personal communications). The list of potential changes to the checklist was assessed in a large, international, online, three-round Delphi survey involving 317 participants and discussed at a two-day online expert consensus meeting of 30 invited international experts.

**Results:**

We have made substantive changes to the CONSORT checklist. We added seven new checklist items, revised three items, deleted one item, and integrated several items from key CONSORT extensions. We also restructured the CONSORT checklist, with a new section on open science. The CONSORT 2025 statement consists of a 30-item checklist of essential items that should be included when reporting the results of a randomised trial and a diagram for documenting the flow of participants through the trial. To facilitate implementation of CONSORT 2025, we have also developed an expanded version of the CONSORT 2025 checklist, with bullet points eliciting critical elements of each item.

**Conclusion:**

Authors, editors, reviewers, and other potential users should use CONSORT 2025 when writing and evaluating manuscripts of randomised trials to ensure that trial reports are clear and transparent.

## Introduction

“Readers should not have to infer what was probably done; they should be told explicitly.” Douglas G Altman 1

Randomised trials, when appropriately designed, conducted, analysed, and reported, are generally considered the highest quality evidence in evaluating healthcare interventions. Critical appraisal of the quality of randomised trials is possible only if their design, conduct, analysis, and results are thoroughly and accurately reported. To interpret a trial accurately, readers need complete and transparent information on its methods and findings. However, extensive evidence displays that the completeness of reporting of randomised trials is inadequate^2^ ^3^ and that incomplete reporting may be associated with biased estimates of intervention effects.^4^ Similarly, having a clear and transparent trial protocol is important because it prespecifies the methods used in the trial, such as the primary outcome, thereby reducing the likelihood of undeclared post hoc changes.^5^

Efforts to improve the reporting of randomised trials gathered impetus in the early 1990s and resulted in the Standardised Reporting of Trials (SORT) and Asilomar initiatives in 1994. Those initiatives then led to publication of the CONSORT (Consolidated Standards of Reporting Trials) statement in 1996,^6^ revised in 2001^7^ with an accompanying explanation and elaboration document.^8^ CONSORT was then updated in 2010,^9^ along with an updated explanation and elaboration article.^10^ Similar problems related to the lack of complete and transparent reporting of trial protocols led to the development of the SPIRIT (Standard Protocol Items: Recommendations for Interventional Trials) statement, published in 2013,^11^ and its accompanying explanation and elaboration document^12^ explaining the principles underlying the statement.

CONSORT is endorsed by numerous journals worldwide and by prominent editorial organisations, including the World Association of Medical Editors (WAME), International Committee of Medical Journal Editors (ICMJE) and Council of Science Editors (CSE). The introduction of CONSORT within journals has been shown to be associated with improved quality of reports of randomised trials. Some evidence shows that journal endorsement of CONSORT is associated with better reporting and that reporting is improving over time.^2^ ^13^ ^14^ ^15^ A Cochrane review of 50 evaluations of 16 604 trials assessed the association between journals’ endorsement of CONSORT and the reporting of trials they published; 25 of 27 CONSORT checklist items were more completely reported when a trial was published in a CONSORT endorsing as opposed to non-endorsing journal.^2^ ^14^ However, a causal effect cannot be proven. At a minimum, CONSORT has sensitised many end users (eg, authors, journal editors, and peer reviewers) to how important careful and thorough reporting can be for randomised trials.

SPIRIT and CONSORT are evidence based guidelines that comprise a checklist of essential items that should be included in protocols and primary reports of completed randomised trials, respectively, and a diagram that documents the flow of participants through a trial. These statements provide guidance to authors on the minimum information that should be included in the reporting of trials to ensure that trial protocols and trial reports are clear and transparent. They are published alongside explanation and elaboration documents, which provide the meaning and rationale for each checklist item, examples of good reporting, and relevant empirical evidence where possible.

In January 2020, the SPIRIT and CONSORT executive groups met in Oxford, UK. As the SPIRIT and CONSORT statements are conceptually linked, with overlapping content and similar dissemination and implementation strategies, the two groups decided it was more effective to work together and formed one group.

**Summary points**

To interpret a randomised trial accurately, readers need complete and transparent information on its methods and findings
The CONSORT 2025 statement provides updated guidance for reporting the results of randomised trials, that reflects methodological advancements and feedback from end users
The CONSORT 2025 statement consists of a 30-item checklist of essential items, a diagram for documenting the flow of participants through the trial, and an expanded checklist that details the critical elements of each checklist item
Authors, editors, reviewers, and other potential users should use CONSORT 2025 when writing and evaluating manuscripts of randomised trials to ensure that trial reports are clear and transparent

## Decision to update the SPIRIT and CONSORT statements

SPIRIT and CONSORT are living guidelines and it is vital that the statements are periodically updated to reflect new evidence, methodological advancements, and feedback from users; otherwise, their value and usefulness will diminish over time.^16^ Updating the SPIRIT 2013 and CONSORT 2010 statements together was also an opportunity to further align both checklists and to provide users with consistent guidance in the reporting of trial design, conduct, analysis, and results from trial protocol to final publication. Harmonising the reporting process should improve usability and adherence, and lead to more-complete reporting.^17^ Here, we introduce the updated CONSORT 2025 statement; the updated SPIRIT 2025 statement is published separately.^18^

## Development of CONSORT 2025

The methods used to update the CONSORT statement followed the EQUATOR Network guidance for developers of health research guidelines^19^ and have been described in detail elsewhere.^20^ ^21^ In brief, we first conducted a scoping review of the literature to identify published comments suggesting modifications and additions or reflecting on strengths and challenges of CONSORT 2010, the findings of which have been published separately.^22^ We also developed a project specific database (SCEBdb) for empirical and theoretical evidence related to CONSORT and risk of bias in randomised trials.^23^ The evidence identified in the scoping review was combined with evidence from, and recommendations provided by the lead authors of, certain key existing CONSORT extensions whose checklist items apply to all trials (Harms,^24^ Outcomes^25^), or a considerable number of trials^26^ (Non-pharmacological Treatment^27^), other related reporting guidelines (the template for intervention description and replication (TIDieR)^28^), and recommendations from other sources (eg, personal communications).

Using the existing CONSORT 2010 checklist as the starting point, a list of potential modifications or additions to the checklists was then created using the gathered evidence from the scoping review and recommendations. This list of potential changes was presented to end users for feedback in a large international online Delphi survey, involving 317 participants who responded to round 1, 303 to round 2 and 290 to round 3. Delphi participants were identified through existing SPIRIT and CONSORT collaborations, and professional research networks and societies. Participants were also recruited via an expression of interest form on the SPIRIT-CONSORT update project website. A broad range of end user roles were represented, the most frequent being statisticians/methodologists/epidemiologists (n=198), systematic reviewers/guideline developers (n=73), trial investigators (n=73), clinicians (n=58), journal editors (n=47), and patient representatives (n=17) (numbers not mutually exclusive). During the three-round Delphi survey, participants were asked to rate on a 5-point Likert scale the extent to which they agreed with the inclusion of each item in the updated CONSORT checklist. Free text boxes were provided for comments on each item and to suggest additional new checklist items.

The Delphi survey results were then presented and discussed at a two-day online expert consensus meeting via Zoom, on 1 and 2 March 2023, attended by 30 invited international participants representing the different stakeholder groups included in the Delphi survey. During the meeting, each new and modified CONSORT checklist item was discussed and agreement sought. An anonymous poll via Zoom was used to help establish the level of support for items where the discussion indicated differing opinions; these polls were advisory and no formal consensus threshold was specified.

After the expert consensus meeting, the executive group held a two-day, in-person writing meeting in Oxford on 25 and 26 April 2023, where the format and wording of each new or modified CONSORT checklist item was reviewed and agreed on. The draft checklist was then circulated to consensus meeting participants to confirm whether they represented the group consensus or needed clarification. CONSORT items were further revised by the executive group in response to this feedback. The finalised items address the minimum content for inclusion in a trial report, although that should not deter prospective authors from including additional information that they deem important or that facilitates replication. Members of the executive group and the 30 invited consensus meeting participants are authors of the manuscript and their names are listed at the end of the manuscript.

## Main changes to CONSORT 2025

We have made a number of substantive changes to the CONSORT 2025 checklist (see box 1). We have added seven new checklist items, revised three items, deleted one item, and integrated several items from key CONSORT extensions (Harms,^24^ Outcomes,^25^ Non-pharmacological Treatment^27^) and other related reporting guidelines (TIDieR 28). We also restructured the CONSORT checklist, with a new section on open science, which includes items that are conceptually linked, such as trial registration (item 2), where the trial protocol and statistical analysis plan can be accessed (item 3), sharing of de-identified participant level data (item 4), and funding and conflicts of interest (item 5). We have also harmonised the wording between CONSORT and SPIRIT checklist items and clarified and simplified the wording of some items. For a detailed comparison of the changes made in the CONSORT 2025 checklist from CONSORT 2010, see appendix 1. We have also updated the CONSORT explanation and elaboration document,^29^ which has been extensively revised and describes the rationale and scientific background for each CONSORT 2025 checklist item and provides published examples of good reporting.

Box 1 **Summary of main changes in CONSORT 2025**

**Addition of new checklist items**

- Item 4: added item on data sharing, including where and how individual de-identified participant data, statistical code, and any other materials can be accessed.
- Item 5b: added item on financial and other conflicts of interest of manuscript authors.
- Item 8: added item on how patients and/or the public were involved in the design, conduct, and/or reporting of the trial.
- Item 12b: added item on eligibility criteria for sites and for individuals delivering the interventions, where applicable
- Item 15: added item on how harms and other unintended effects were assessed.
- Item 21: added items to define who is included in each analysis (eg, all randomised participants) and in which group (item 21b), and how missing data were handled in the analysis (item 21c).
- Item 24: added item on intervention delivery, including how the intervention and comparator were actually administered (item 24a) and details of concomitant care received during the trial (item 24b).

**Completely revised checklist items**

- Item 3: revised item to include where the statistical analysis plan can be accessed in addition to the trial protocol.
- Item 10: revised item to include reporting of important changes to the trial after it commenced, including any outcomes or analyses that were not prespecified.
- Item 26: revised item to specify for each primary and secondary outcome—the number of participants included in the analysis and the number of participants with available data at each time point for each treatment group.

**Deletion of checklist item**

- Deleted item on generalisability of trial findings, which is now incorporated under trial limitations (item 30).

**Integration of checklist items from key CONSORT extensions**

- Addition of items related to reporting of how harms^24^ were assessed and analysed (items 7, 15, 21a, 23a, 27), how outcomes^25^ were measured and analysed (items 14, 26), and how the intervention^27^ ^28^ and comparator were actually administered and by whom (item 24).

**Structure and organisation of checklist items**

- Restructuring of checklist, with a new section on open science, which includes items that are conceptually linked such as trial registration (item 2), where the trial protocol and statistical analysis plan can be accessed (item 3), sharing of de-identified participant level data (item 4), and funding and conflicts of interest (item 5).
- Aligned wording of some CONSORT checklist items with that of SPIRIT checklist items and vice versa.
- Clarified and simplified wording of some items.

To help facilitate implementation of CONSORT 2025, we have also developed an expanded version of the CONSORT 2025 checklist, with bullet points eliciting critical elements of each item. This is similar to the model proposed by the COBWEB (CONSORT-based web tool)^30^ and COBPeer (CONSORT based peer review tool)^31^ studies and used in the 2020 PRISMA guidance for reporting systematic reviews.^32^ The expanded checklist comprises an abridged version of elements presented in the CONSORT 2025 explanation and elaboration document,^29^ with examples and references removed (see appendix 2).

## Scope of CONSORT 2025

**Fig 1:**
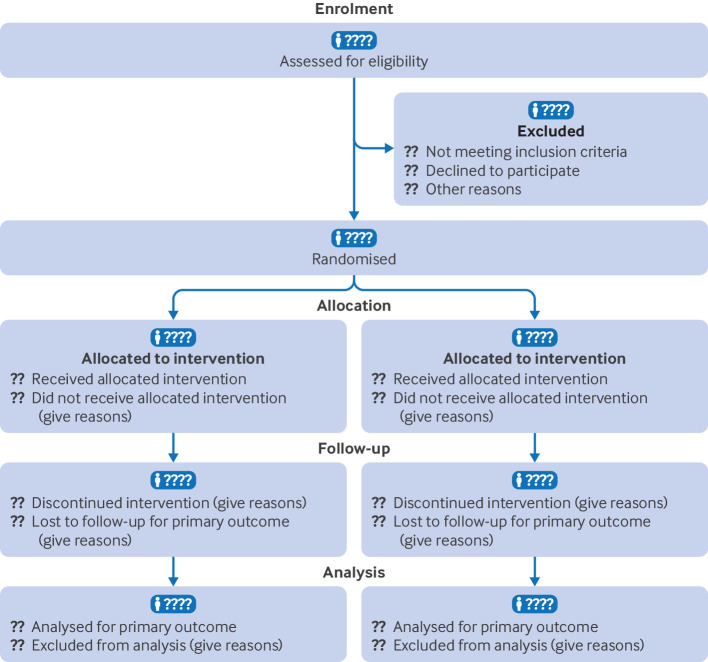
CONSORT 2025 flow diagram. Flow diagram of the progress through the phases of a randomised trial of two groups (ie, enrolment, intervention allocation, follow-up, and data analysis). CONSORT=Consolidated Standards of Reporting Trials

The CONSORT 2025 statement comprises a 30-item checklist and provides a minimum set of items to be included in a report of a randomised trial (table 1) and a diagram for documenting the flow of participants through a trial (fig 1). We strongly recommend the CONSORT 2025 statement be used alongside the CONSORT 2025 explanation and elaboration document.^29^ The CONSORT 2025 statement supersedes the CONSORT 2010 statement, which should no longer be used. Journal editors and publishers should update their instructions to authors to refer to CONSORT 2025. CONSORT 2025 provides guidance for reporting all randomised trials but focuses on the most common type, the two-group parallel design.

**Table 1:** CONSORT 2025 checklist of information to include when reporting a randomised trial

Section/topic	No	CONSORT 2025 checklist item description
**Title and abstract**		
Title and structured abstract	1a	Identification as a randomised trial
	1b	Structured summary of the trial design, methods, results, and conclusions
**Open science**		
Trial registration	2	Name of trial registry, identifying number (with URL) and date of registration
Protocol and statistical analysis plan	3	Where the trial protocol and statistical analysis plan can be accessed
Data sharing	4	Where and how the individual de-identified participant data (including data dictionary), statistical code and any other materials can be accessed
Funding and conflicts of interest	5a	Sources of funding and other support (eg, supply of drugs), and role of funders in the design, conduct, analysis and reporting of the trial
	5b	Financial and other conflicts of interest of the manuscript authors
**Introduction**		
Background and rationale	6	Scientific background and rationale
Objectives	7	Specific objectives related to benefits and harms
**Methods**		
Patient and public involvement	8	Details of patient or public involvement in the design, conduct and reporting of the trial
Trial design	9	Description of trial design including type of trial (eg, parallel group, crossover), allocation ratio, and framework (eg, superiority, equivalence, non-inferiority, exploratory)
Changes to trial protocol	10	Important changes to the trial after it commenced including any outcomes or analyses that were not prespecified, with reason
Trial setting	11	Settings (eg, community, hospital) and locations (eg, countries, sites) where the trial was conducted
Eligibility criteria	12a	Eligibility criteria for participants
	12b	If applicable, eligibility criteria for sites and for individuals delivering the interventions (eg, surgeons, physiotherapists)
Intervention and comparator	13	Intervention and comparator with sufficient details to allow replication. If relevant, where additional materials describing the intervention and comparator (eg, intervention manual) can be accessed
Outcomes	14	Prespecified primary and secondary outcomes, including the specific measurement variable (eg, systolic blood pressure), analysis metric (eg, change from baseline, final value, time to event), method of aggregation (eg, median, proportion), and time point for each outcome
Harms	15	How harms were defined and assessed (eg, systematically, non-systematically)
Sample size	16a	How sample size was determined, including all assumptions supporting the sample size calculation
	16b	Explanation of any interim analyses and stopping guidelines
Randomisation:		
Sequence generation	17a	Who generated the random allocation sequence and the method used
	17b	Type of randomisation and details of any restriction (eg, stratification, blocking and block size)
Allocation concealment mechanism	18	Mechanism used to implement the random allocation sequence (eg, central computer/telephone; sequentially numbered, opaque, sealed containers), describing any steps to conceal the sequence until interventions were assigned
Implementation	19	Whether the personnel who enrolled and those who assigned participants to the interventions had access to the random allocation sequence
Blinding	20a	Who was blinded after assignment to interventions (eg, participants, care providers, outcome assessors, data analysts)
	20b	If blinded, how blinding was achieved and description of the similarity of interventions
Statistical methods	21a	Statistical methods used to compare groups for primary and secondary outcomes, including harms
	21b	Definition of who is included in each analysis (eg, all randomised participants), and in which group
	21c	How missing data were handled in the analysis
	21d	Methods for any additional analyses (eg, subgroup and sensitivity analyses), distinguishing prespecified from post hoc
**Results**		
Participant flow, including flow diagram	22a	For each group, the numbers of participants who were randomly assigned, received intended intervention, and were analysed for the primary outcome
	22b	For each group, losses and exclusions after randomisation, together with reasons
Recruitment	23a	Dates defining the periods of recruitment and follow-up for outcomes of benefits and harms
	23b	If relevant, why the trial ended or was stopped
Intervention and comparator delivery	24a	Intervention and comparator as they were actually administered (eg, where appropriate, who delivered the intervention/comparator, how participants adhered, whether they were delivered as intended (fidelity))
	24b	Concomitant care received during the trial for each group
Baseline data	25	A table showing baseline demographic and clinical characteristics for each group
Numbers analysed, outcomes and estimation	26	For each primary and secondary outcome, by group: ● the number of participants included in the analysis ● the number of participants with available data at the outcome time point ● result for each group, and the estimated effect size and its precision (such as 95% confidence interval) ● for binary outcomes, presentation of both absolute and relative effect size
Harms	27	All harms or unintended events in each group
Ancillary analyses	28	Any other analyses performed, including subgroup and sensitivity analyses, distinguishing pre-specified from post hoc
**Discussion**		
Interpretation	29	Interpretation consistent with results, balancing benefits and harms, and considering other relevant evidence
Limitations	30	Trial limitations, addressing sources of potential bias, imprecision, generalisability, and, if relevant, multiplicity of analyses

Extensions to CONSORT have been developed to tackle the methodological issues associated with reporting different types of trial designs, data, and interventions. Examples of extensions for trial designs include recommendations for adaptive designs,^33^ cluster trials,^34^ crossover trials,^35^ early phase trials,^36^ factorial trials,^37^ non-inferiority and equivalence trials,^38^ pragmatic trials,^39^ multi-arm trials,^40^ n-of-1 trials,^41^ pilot and feasibility trials,^42^ and within-person trials.^43^ Other extensions include non-pharmacological treatments,^27^ outcomes,^25^ patient reported outcomes,^44^ surrogate outcomes,^45^ social and psychological interventions,^46^ harms,^24^ abstracts,^47^ and health equity.^48^ We will engage with the leaders of these extensions to implement a process for aligning them with the updated CONSORT 2025 statement. In the meantime, we recommend that readers use the existing version of the relevant CONSORT extension(s).

## Implication and limitations

The objective of the CONSORT 2025 statement is to provide a minimum set of recommendations to authors about the content they should include in order to report their trials in a clear, complete, and transparent manner.^9^ ^10^ Readers, peer reviewers, clinicians, guideline writers, patients and the public, and editors can also use CONSORT 2025 to help them appraise the reporting of randomised trials. We also strongly recommend the submission of a completed CONSORT 2025 checklist as part of the manuscript submission process, detailing where in the manuscript checklist items are reported, and uploaded as part of the supplementary materials.^49^ An explicit description of what was done and what was found, without ambiguity or omission, best serves the interests of all readers.^9^

It is important to note that CONSORT 2025 and SPIRIT 2025 do not include recommendations for designing, conducting, or analysing trials, but nevertheless the recommendations contained here can help researchers in the design, conduct, and analysis of their trial by highlighting key issues to consider. Updating the SPIRIT and CONSORT statements together was also an opportunity to align reporting in both checklists and to provide users with consistent guidance in the reporting of trial design, conduct, and analysis, from the trial protocol to final publication.^17^ Thus, clear and transparent reports of trial protocols should in turn facilitate properly designed and well conducted trials. In addition, transparent reporting of trial results can reveal deficiencies in research if they exist and allow better estimates of their prevalence and severity. Importantly, however, CONSORT 2025 is not meant to be used as a quality assessment instrument. Rather, the content of CONSORT 2025 focuses on reporting items related to the internal and external validity of randomised trials.

With CONSORT 2025, we do not suggest a rigid structure for the reporting of randomised trials. Instead, the format of articles should abide by the journal’s individual style and its “Instructions to Authors.” Authors should address checklist items somewhere in the article, with sufficient detail and clarity.^9^ We also promote the use of additional online supplementary material to allow for more detailed reporting of the trial methods and results than may be permissible within the typical length of some print journal articles. Full data and code sharing offers another, higher level of transparency and we recommend providing detailed information on whether this is happening or planned to happen (eg, after some time) in a randomised trial.

CONSORT urges clarity and transparency of reporting which reflects the actual trial design, conduct, and analysis. High quality reporting is an important step when considering issues related to reproducibility.^50^ We encourage trial authors to detail what was done and to acknowledge if something was not done or was modified, ensuring alignment of information with that reported in the trial protocol, statistical analysis plan, and trial registry. A joint SPIRIT-CONSORT website (https://www.consort-spirit.org/) has been established to provide more information about the CONSORT and SPIRIT statements, including additional resources and training materials aimed at researchers, research trainees, journal editors, and peer reviewers. The website also includes resources aimed at patients and the public that explain the importance of clear and transparent reporting of randomised trials and their importance in the delivery of evidence based healthcare.

CONSORT 2025 represents a living guideline that will continue to be periodically updated to reflect new evidence and emerging perspectives. Such an approach is important to ensure the guidance remains relevant to end users, including authors, patients and the public, journal editors, and peer reviewers.

## Web extra

Extra material supplied by authors

Web appendix 1: Comparison of CONSORT 2025 and CONSORT 2010 checklists

Web appendix 2: CONSORT 2025 expanded checklist of detailed information to include when reporting a randomised trial

## Notes

Contributors: SH, IB, A-WC, AH, KFS, and DM were responsible for the concept; SH, IB, A-WC, GSC, AH, KFS, and DM were responsible for the funding. SH, IB, A-WC, GSC, AH, KFS, RT, and DM drafted specific sections of the manuscript. All authors then critically revised the manuscript for important intellectual content and approved the final version. SH is the guarantor of this work. The corresponding author attests that all listed authors meet authorship criteria and that no others meeting the criteria have been omitted.

Patient and public involvement: The CONSORT 2025 checklist items and the explanations here were developed using input from an international Delphi survey and consensus meeting. The Delphi survey was advertised via established patient and public involvement (PPI) networks, and 17 respondents self-identified as a “patient or public representative” and completed the Delphi survey. In addition, three of the participants in the expert consensus meeting were patient or public representatives who were leaders in advancing PPI.

Dissemination to participants and related patient and public communities: CONSORT 2025 will be disseminated via a new website, consort-spirit.org, which will include materials designed for patients and the public.

Provenance and peer review: Not commissioned; externally peer reviewed.

## References

[1] Altman DG . Better reporting of randomised controlled trials: the CONSORT statement. BMJ 1996;313:570-1. 10.1136/bmj.313.7057.570 8806240 PMC2352018

[2] Turner L Shamseer L Altman DG Schulz KF Moher D . Does use of the CONSORT Statement impact the completeness of reporting of randomised controlled trials published in medical journals? A Cochrane review. Syst Rev 2012;1:60. 10.1186/2046-4053-1-60 23194585 PMC3564748

[3] Glasziou P Altman DG Bossuyt P . Reducing waste from incomplete or unusable reports of biomedical research. Lancet 2014;383:267-76. 10.1016/S0140-6736(13)62228-X 24411647

[4] Savović J Jones HE Altman DG . Influence of reported study design characteristics on intervention effect estimates from randomized, controlled trials. Ann Intern Med 2012;157:429-38. 10.7326/0003-4819-157-6-201209180-00537 22945832

[5] COMPARE. Tracking switched outcomes in clinical trials. https://www.compare-trials.org/ [accessed 26 May 2021].

[6] Begg C Cho M Eastwood S . Improving the quality of reporting of randomized controlled trials. The CONSORT statement. JAMA 1996;276:637-9. 10.1001/jama.1996.03540080059030 8773637

[7] Moher D Schulz KF Altman D CONSORT Group (Consolidated Standards of Reporting Trials) . The CONSORT statement: revised recommendations for improving the quality of reports of parallel-group randomized trials. JAMA 2001;285:1987-91. 10.1001/jama.285.15.1987 11308435

[8] Altman DG Schulz KF Moher D CONSORT GROUP (Consolidated Standards of Reporting Trials) . The revised CONSORT statement for reporting randomized trials: explanation and elaboration. Ann Intern Med 2001;134:663-94. 10.7326/0003-4819-134-8-200104170-00012 11304107

[9] Schulz KF Altman DG Moher D CONSORT Group . CONSORT 2010 statement: updated guidelines for reporting parallel group randomized trials. Ann Intern Med 2010;152:726-32. 10.7326/0003-4819-152-11-201006010-00232 20335313

[10] Moher D Hopewell S Schulz KF . CONSORT 2010 explanation and elaboration: updated guidelines for reporting parallel group randomised trials. BMJ 2010;340:c869. 10.1136/bmj.c869 20332511 PMC2844943

[11] Chan AW Tetzlaff JM Altman DG . SPIRIT 2013 statement: defining standard protocol items for clinical trials. Ann Intern Med 2013;158:200-7. 10.7326/0003-4819-158-3-201302050-00583 23295957 PMC5114123

[12] Chan A-W Tetzlaff JM Gøtzsche PC . SPIRIT 2013 explanation and elaboration: guidance for protocols of clinical trials. BMJ 2013;346:e7586. 10.1136/bmj.e7586 23303884 PMC3541470

[13] Plint AC Moher D Morrison A . Does the CONSORT checklist improve the quality of reports of randomised controlled trials? A systematic review. Med J Aust 2006;185:263-7. 10.5694/j.1326-5377.2006.tb00557.x 16948622

[14] Dechartres A Trinquart L Atal I . Evolution of poor reporting and inadequate methods over time in 20 920 randomised controlled trials included in Cochrane reviews: research on research study. BMJ 2017;357:j2490. 10.1136/bmj.j2490 28596181

[15] Moher D Jones A Lepage L CONSORT Group (Consolidated Standards for Reporting of Trials) . Use of the CONSORT statement and quality of reports of randomized trials: a comparative before-and-after evaluation. JAMA 2001;285:1992-5. 10.1001/jama.285.15.1992 11308436

[16] Simera I Altman DG . ACP Journal Club. Editorial: Writing a research article that is “fit for purpose”: EQUATOR Network and reporting guidelines. Ann Intern Med 2009;151:JC2-2, JC2-3. 10.7326/0003-4819-151-4-200908180-02002 19687477

[17] Hopewell S Boutron I Chan AW . An update to SPIRIT and CONSORT reporting guidelines to enhance transparency in randomized trials. Nat Med 2022;28:1740-3. 10.1038/s41591-022-01989-8 36109642

[18] Chan A-W Boutron I Hopewell S . SPIRIT 2025 statement: updated guideline for protocols of randomised trials. BMJ 2025;389:e081477. 10.1136/bmj-2024-081477 PMC12035670 40294953

[19] Moher D Schulz KF Simera I Altman DG . Guidance for developers of health research reporting guidelines. PLoS Med 2010;7:e1000217. 10.1371/journal.pmed.1000217 20169112 PMC2821895

[20] Hopewell S, Chan AW, Boutron I, et al. Protocol for updating the SPIRIT 2013 (Standard Protocol Items: Recommendations for Interventional Trials) and CONSORT 2010 (CONsolidated Standards Of Reporting Trials) Statements (version 1.0, August 2022). 10.17605/OSF.IO/6HJYG. 2022.

[21] Tunn R Boutron I Chan A-W . Methods used to develop the SPIRIT 2024 and CONSORT 2024 Statements. J Clin Epidemiol 2024;169:111309. 10.1016/j.jclinepi.2024.111309 38428538

[22] Nejstgaard CH Boutron I Chan AW . A scoping review identifies multiple comments suggesting modifications to SPIRIT 2013 and CONSORT 2010. J Clin Epidemiol 2023;155:48-63. 10.1016/j.jclinepi.2023.01.003 36669708

[23] Østengaard L Barrientos A Boutron I . Development of a topic-specific bibliographic database supporting the updates of SPIRIT 2013 and CONSORT 2010. Cochrane Evidence Synthesis and Methods 2024;2:e12057 10.1002/cesm.12057. PMC11795946 40476263

[24] Junqueira DR Zorzela L Golder S CONSORT Harms Group . CONSORT Harms 2022 statement, explanation, and elaboration: updated guideline for the reporting of harms in randomized trials. J Clin Epidemiol 2023;158:149-65. 10.1016/j.jclinepi.2023.04.005 37100738

[25] Butcher NJ Monsour A Mew EJ . Guidelines for reporting outcomes in trial reports: the CONSORT-Outcomes 2022 extension. JAMA 2022;328:2252-64. 10.1001/jama.2022.21022 36511921

[26] Ghosn L Boutron I Ravaud P . Consolidated Standards of Reporting Trials (CONSORT) extensions covered most types of randomized controlled trials, but the potential workload for authors was high. J Clin Epidemiol 2019;113:168-75. 10.1016/j.jclinepi.2019.05.030 31153976

[27] Boutron I Altman DG Moher D Schulz KF Ravaud P CONSORT NPT Group . CONSORT statement for randomized trials of nonpharmacologic treatments: a 2017 update and a CONSORT extension for nonpharmacologic trial abstracts. Ann Intern Med 2017;167:40-7. 10.7326/M17-0046 28630973

[28] Hoffmann TC Glasziou PP Boutron I . Better reporting of interventions: template for intervention description and replication (TIDieR) checklist and guide. BMJ 2014;348:g1687. 10.1136/bmj.g1687 24609605

[29] Hopewell S Chan A-W Collins GS . CONSORT 2025 explanation and elaboration: updated guideline for reporting randomised trials. BMJ 2025;389:e081124. 10.1136/bmj-2024-081124 PMC11995452 40228832

[30] Barnes C Boutron I Giraudeau B Porcher R Altman DG Ravaud P . Impact of an online writing aid tool for writing a randomized trial report: the COBWEB (Consort-based WEB tool) randomized controlled trial. BMC Med 2015;13:221. 10.1186/s12916-015-0460-y 26370288 PMC4570037

[31] Chauvin A Ravaud P Moher D . Accuracy in detecting inadequate research reporting by early career peer reviewers using an online CONSORT-based peer-review tool (COBPeer) versus the usual peer-review process: a cross-sectional diagnostic study. BMC Med 2019;17:205. 10.1186/s12916-019-1436-0 31744489 PMC6864983

[32] Page MJ McKenzie JE Bossuyt PM . The PRISMA 2020 statement: an updated guideline for reporting systematic reviews. Syst Rev 2021;10:89. 10.1186/s13643-021-01626-4 33781348 PMC8008539

[33] Dimairo M Pallmann P Wason J ACE Consensus Group . The adaptive designs CONSORT extension (ACE) statement: a checklist with explanation and elaboration guideline for reporting randomised trials that use an adaptive design. Trials 2020;21:528. 10.1186/s13063-020-04334-x 32546273 PMC7298968

[34] Campbell MK Piaggio G Elbourne DR Altman DG CONSORT Group . Consort 2010 statement: extension to cluster randomised trials. BMJ 2012;345:e5661. 10.1136/bmj.e5661 22951546

[35] Dwan K Li T Altman DG Elbourne D . CONSORT 2010 statement: extension to randomised crossover trials. BMJ 2019;366:l4378. 10.1136/bmj.l4378 31366597 PMC6667942

[36] Yap C Solovyeva O de Bono J . Enhancing reporting quality and impact of early phase dose-finding clinical trials: CONSORT Dose-finding Extension (CONSORT-DEFINE) guidance. BMJ 2023;383:e076387. 10.1136/bmj-2023-076387 37863501 PMC10583500

[37] Kahan BC Hall SS Beller EM . Reporting of factorial randomized trials: extension of the CONSORT 2010 statement. JAMA 2023;330:2106-14. 10.1001/jama.2023.19793 38051324 PMC7617336

[38] Piaggio G Elbourne DR Pocock SJ Evans SJW Altman DG CONSORT Group . Reporting of noninferiority and equivalence randomized trials: extension of the CONSORT 2010 statement. JAMA 2012;308:2594-604. 10.1001/jama.2012.87802 23268518

[39] Zwarenstein M Treweek S Gagnier JJ CONSORT group Pragmatic Trials in Healthcare (Practihc) group . Improving the reporting of pragmatic trials: an extension of the CONSORT statement. BMJ 2008;337:a2390. 10.1136/bmj.a2390 19001484 PMC3266844

[40] Juszczak E Altman DG Hopewell S Schulz K . Reporting of multi-arm parallel-group randomized trials: extension of the CONSORT 2010 statement. JAMA 2019;321:1610-20. 10.1001/jama.2019.3087 31012939

[41] Vohra S Shamseer L Sampson M CENT Group . CONSORT extension for reporting N-of-1 trials (CENT) 2015 Statement. BMJ 2015;350:h1738. 10.1136/bmj.h1738. 25976398

[42] Eldridge SM Chan CL Campbell MJ PAFS consensus group . CONSORT 2010 statement: extension to randomised pilot and feasibility trials. BMJ 2016;355:i5239. 10.1136/bmj.i5239 27777223 PMC5076380

[43] Pandis N Chung B Scherer RW Elbourne D Altman DG . CONSORT 2010 statement: extension checklist for reporting within person randomised trials. BMJ 2017;357:j2835. 10.1136/bmj.j2835 28667088 PMC5492474

[44] Calvert M Blazeby J Altman DG Revicki DA Moher D Brundage MD CONSORT PRO Group . Reporting of patient-reported outcomes in randomized trials: the CONSORT PRO extension. JAMA 2013;309:814-22. 10.1001/jama.2013.879 23443445

[45] Manyara AM Davies P Stewart D . Reporting of surrogate endpoints in randomised controlled trial reports (CONSORT-Surrogate): extension checklist with explanation and elaboration. BMJ 2024;386:e078524. 10.1136/bmj-2023-078524 38981645 PMC11231881

[46] Montgomery P Grant S Mayo-Wilson E CONSORT-SPI Group . Reporting randomised trials of social and psychological interventions: the CONSORT-SPI 2018 Extension. Trials 2018;19:407. 10.1186/s13063-018-2733-1 30060754 PMC6066921

[47] Hopewell S Clarke M Moher D CONSORT Group . CONSORT for reporting randomized controlled trials in journal and conference abstracts: explanation and elaboration. PLoS Med 2008;5:e20. 10.1371/journal.pmed.0050020 18215107 PMC2211558

[48] Welch VA Norheim OF Jull J Cookson R Sommerfelt H Tugwell P CONSORT-Equity and Boston Equity Symposium . CONSORT-Equity 2017 extension and elaboration for better reporting of health equity in randomised trials. BMJ 2017;359:j5085. 10.1136/bmj.j5085 29170161

[49] Hirst A Altman DG . Are peer reviewers encouraged to use reporting guidelines? A survey of 116 health research journals. PLoS One 2012;7:e35621. 10.1371/journal.pone.0035621 22558178 PMC3338712

[50] Goodman SN Fanelli D Ioannidis JP . What does research reproducibility mean? Sci Transl Med 2016;8:341ps12. 10.1126/scitranslmed.aaf5027 27252173

