# Supplementary material for: CONSORT 2025 statement: updated guideline for reporting randomised trials
Source: BMJ. 2025 Apr 14;389:e081123. doi: 10.1136/bmj-2024-081123 (PMC11995449; doi:10.1136/bmj-2024-081123)
Supplement: Supplementary file 1 — Web appendix 1: Comparison of CONSORT 2025 and CONSORT 2010 checklists [file hops081123.ww1.pdf]

Appendix 1: Comparison of CONSORT 2025 and CONSORT 2010 checklists

Cite as: Hopewell S, Chan A-W, Collins GS, et al. CONSORT 2025 statement: updated guideline for reporting randomised trials. *BMJ* 2025;389:e081123.

doi:10.1136/bmj-2024-081123

**CONSORT 2025**			***CONSORT 2010***		
**Section / Topic**	**No**	**CONSORT 2025 checklist item**	***Section/Topic***	***No***	***CONSORT 2010 checklist item***
**Title and abstract**			***Title and abstract***		
Title and structured abstract	1a	Identification as a randomised trial	*Title and abstract*	*1a*	*Identification as a randomised trial in the title*
	1b	Structured summary of the trial design, methods, results, and conclusions		*1b*	*Structured summary of trial design, methods, results, and conclusions (for specific guidance see CONSORT for abstracts)*
**Open science**			***Other information***		
Trial registration	2	Name of trial registry, identifying number (with URL) and date of registration	*Registration*	*23*	*Registration number and name of trial registry*
Protocol and statistical analysis plan	3	Where the trial protocol and statistical analysis plan can be accessed	*Protocol*	*24*	*Where the full trial protocol can be accessed, if available*
Data sharing	4	Where and how the individual de-identified participant data (including data dictionary), statistical code and any other materials can be accessed			
Funding and conflicts of interest	5a	Sources of funding and other support (e.g., supply of drugs), and role of funders in the design, conduct, analysis and reporting of the trial	*Funding*	*25*	*Sources of funding and other support (such as supply of drugs), role of funders*
	5b	Financial and other conflicts of interest of the manuscript authors			
**Introduction**			***Introduction***		
Background and rationale	6	Scientific background and rationale	*Background and objectives*	*2a*	*Scientific background and explanation of rationale*
Objectives	7	Specific objectives related to benefits and harms		*2b*	*Specific objectives or hypotheses*
**Methods**			***Methods***		
Patient and public involvement	8	Details of patient or public involvement in the design, conduct and reporting of the trial			

Trial design	9	Description of trial design including type of trial (e.g., parallel group, crossover), allocation ratio, and framework (e.g., superiority, equivalence, non-inferiority, exploratory)	*Trial design*	*3a*	*Description of trial design (such as parallel, factorial) including allocation ratio*
Changes to trial protocol	10	Important changes to the trial after it commenced including any outcomes or analyses that were not prespecified, with reason		*3b*	*Important changes to methods after trial commencement (such as eligibility criteria), with reasons*
			Outcomes	*6b*	*Any changes to trial outcomes after the trial commenced, with reasons*
Trial setting	11	Settings (e.g., community, hospital) and locations (e.g., countries, sites) where the trial was conducted	*Participants*	*4b*	*Settings and locations where the data were collected*
Eligibility criteria	12a	Eligibility criteria for participants		*4a*	*Eligibility criteria for participants*
	12b	If applicable, eligibility criteria for sites and for individuals delivering the interventions (e.g., surgeons, physiotherapists)			
Intervention and comparator	13	Intervention and comparator with sufficient details to allow replication. If relevant, where additional materials describing the intervention and comparator (e.g., intervention manual) can be accessed	*Interventions*	*5*	*The interventions for each group with sufficient details to allow replication, including how and when they were actually administered*
Outcomes	14	Pre-specified primary and secondary outcomes, including the specific measurement variable (e.g., systolic blood pressure), analysis metric (e.g., change from baseline, final value, time to event), method of aggregation (e.g., median, proportion), and time point for each outcome	*Outcomes*	*6a*	*Completely defined pre-specified primary and secondary outcome measures, including how and when they were assessed*
Harms	15	How harms were defined and assessed (e.g., systematically, non-systematically)			
Sample size	16a	How sample size was determined, including all assumptions supporting the sample size calculation	*Sample size*	*7a*	*How sample size was determined*
	16b	Explanation of any interim analyses and stopping guidelines		*7b*	*When applicable, explanation of any interim analyses and stopping guidelines*
Randomisation:			*Randomisation:*		
Sequence generation	17a	Who generated the random allocation sequence and the method used	*Implementation*	*10**	*Who generated the random allocation sequence, [who enrolled participants, and who assigned participants to interventions]*
			Sequence generation	*8a*	*Method used to generate the random allocation sequence*

	17b	Type of randomisation and details of any restriction (e.g., stratification, blocking and block size)	*Sequence generation*	*8b*	*Type of randomisation; details of any restriction (such as blocking and block size)*
Allocation concealment mechanism	18	Mechanism used to implement the random allocation sequence (e.g., central computer/telephone; sequentially numbered, opaque, sealed containers), describing any steps to conceal the sequence until interventions were assigned	*Allocation concealment mechanism*	*9*	*Mechanism used to implement the random allocation sequence (such as sequentially numbered containers),*<br>*describing any steps taken to conceal the sequence until interventions were assigned*
Implementation	19	Whether the personnel who enrolled and those who assigned participants to the interventions had access to the random allocation sequence	*Implementation*	*10**	*[Who generated the random allocation sequence,] who enrolled participants, and who assigned participants to interventions*
Blinding	20a	Who was blinded after assignment to interventions (e.g., participants, care providers, outcome assessors, data analysts)	*Blinding*	*11a*	*If done, who was blinded after assignment to interventions (for example, participants, care providers, those assessing outcomes) and how*
	20b	If blinded, how blinding was achieved and description of the similarity of interventions		*11b*	*If relevant, description of the similarity of interventions*
Statistical methods	21a	Statistical methods used to compare groups for primary and secondary outcomes, including harms	*Statistical methods*	*12a*	*Statistical methods used to compare groups for primary and secondary outcomes*
	21b	Definition of who is included in each analysis (e.g., all randomised participants), and in which group			
	21c	How missing data were handled in the analysis			
	21d	Methods for any additional analyses (e.g., subgroup and sensitivity analyses), distinguishing prespecified from post-hoc		*12b*	*Methods for additional analyses, such as subgroup analyses and adjusted analyses*
**Results**			***Results***		
Participant flow, including flow diagram	22a	For each group, the numbers of participants who were randomly assigned, received intended intervention, and were analysed for the primary outcome	*Participant flow (a diagram is strongly recommended)*	*13a*	*For each group, the numbers of participants who were randomly assigned, received intended treatment, and*<br>*were analysed for the primary outcome*
	22b	For each group, losses and exclusions after randomisation, together with reasons		*13b*	*For each group, losses and exclusions after randomisation, together with reasons*
Recruitment	23a	Dates defining the periods of recruitment and follow-up for outcomes of benefits and harms	*Recruitment*	*14a*	*Dates defining the periods of recruitment and follow-up*
	23b	If relevant, why the trial ended or was stopped		*14b*	*Why the trial ended or was stopped*
Intervention and comparator delivery	24a	Intervention and comparator as they were actually administered (e.g., where appropriate, who delivered the			

		intervention/comparator, how participants adhered, whether they were delivered as intended [fidelity])			
	24b	Concomitant care received during the trial for each group			
Baseline data	25	A table showing baseline demographic and clinical characteristics for each group	*Baseline data*	*15*	*A table showing baseline demographic and clinical characteristics for each group*
Numbers analysed, outcomes and estimation	26	For each primary and secondary outcome, by group:<br>● the number of participants included in the analysis<br>● the number of participants with available data at the outcome time point<br>● result for each group, and the estimated effect size and its precision (such as 95% confidence interval)<br>● for binary outcomes, presentation of both absolute and relative effect size	*Numbers analysed*	*16*	*For each group, number of participants (denominator) included in each analysis and whether the analysis was by original assigned groups*
			Outcomes and estimation	*17a*	*For each primary and secondary outcome, results for each group, and the estimated effect size and its precision (such as 95% confidence interval)*
				17b	*For binary outcomes, presentation of both absolute and relative effect sizes is recommended*
Harms	27	All harms or unintended events in each group	*Harms*	*19*	*All important harms or unintended effects in each group (for specific guidance see CONSORT for harms)*
Ancillary analyses	28	Any other analyses performed, including subgroup and sensitivity analyses, distinguishing pre-specified from post-hoc	*Ancillary analyses*	*18*	*Results of any other analyses performed, including subgroup analyses and adjusted analyses, distinguishing pre-specified from exploratory*
**Discussion**			***Discussion***		
Interpretation	29	Interpretation consistent with results, balancing benefits and harms, and considering other relevant evidence	*Interpretation*	*22*	*Interpretation consistent with results, balancing benefits and harms, and considering other relevant evidence*
Limitations	30	Trial limitations, addressing sources of potential bias, imprecision, generalisability, and, if relevant, multiplicity of analyses	*Limitations*	*20*	*Trial limitations, addressing sources of potential bias, imprecision, and, if relevant, multiplicity of analyses*
			Generalisability	*21*	*Generalisability (external validity, applicability) of the trial findings*

*Item 10 in CONSORT 2010 has been split and is included partly in item 17a and partly in item 19 in CONSORT 2024
